# Decisive evidence corroborates a null relationship between MTHFR C677T and chronic kidney disease: A case–control study and a meta-analysis

**DOI:** 10.1097/MD.0000000000021045

**Published:** 2020-07-17

**Authors:** Hsueh-Lu Chang, Guei-Rung Chen, Po-Jen Hsiao, Chih-Chien Chiu, Ming-Cheng Tai, Chung-Cheng Kao, Dung-Jang Tsai, Hao Su, Yu-Hsuan Chen, Wei-Teing Chen, Sui-Lung Su

**Affiliations:** a School of Public Health; b School of Dentistry; c Center for General Education, National Defense Medical Center, Taipei; d Department of Internal Medicine, Taoyuan Armed Forces General Hospital; e Division of Infectious Diseases, Department of Internal Medicine, Taoyuan Armed Forces General Hospital, National Defense Medical Center, Taoyuan; f Department of Ophthalmology, Tri-Service General Hospital, National Defense Medical Center, Taipei; g Department of Emergency Medicine, Taoyuan Armed Forces General Hospital, National Defense Medical Center, Taoyuan; h Graduate Institute of Life Sciences, National Defense Medical Center, Taipei; i Department of Health Industry Management, Kainan University, Taoyuan; j Division of Thoracic Medicine, Department of Medicine, Cheng Hsin General Hospital; k Department of Medicine, Tri-Service General Hospital, National Defense Medical Center, Taipei, Taiwan, ROC.

**Keywords:** case–control study, chronic kidney disease, gene polymorphism, gene–environment interaction, meta-analysis, meta-regression, *methylenetetrahydrofolate reductase*, trial sequential analysis

## Abstract

**Background:**

Previous meta-analyses have explored the association between the C677T polymorphism of *methyltetrahydrofolate reductase* (*MTHFR)* and chronic kidney disease (CKD) but there were no studies with a decisive conclusion. Furthermore, the high heterogeneity among different populations is not yet interpreted.

**Objectives:**

This study used trial sequential analysis (TSA) to evaluate whether the nowadays conclusion supported by current cumulative samples. We also applied case-weighted meta-regression to explore the potential gene–environment interactions.

**Methods:**

For the first stage of this study we conducted a case–control study involving 847 dialysis patients from 7 hemodialysis centers in Taipei during 2015 to 2018 and 755 normal controls from a health center in the Tri-Service General Hospital. The second stage combined the results from the first stage with previous studies. The previous studies were collected from PubMed, EMBASE, and Web of Science databases before January 2018.

**Results:**

From the case–control study, the T allele of *MTHFR* C677T appeared to have a protective effect on end-stage renal disease compared with the C allele [odds ratio (OR): 0.80, 95% CI (confidence interval) = 0.69–0.93]. However, the meta-analysis contradicted the results in Asian (OR = 1.12, 95% CI = 0.96–1.30). The same analysis was also applied in Caucasian and presented similar results from Asian (OR = 1.18, 95% CI = 0.98–1.42). The TSA showed our case–control study to be the decisive sample leading to a null association among Asian population. The high heterogeneity (I^2^ = 75%) could explain the contradictory results between the case–control study and the meta-analysis. However, further case-weighted meta-regression did not find any significant interaction between measured factors and *MTHFR* C677T on CKD.

**Conclusions:**

High heterogeneities were found in both Caucasian and Asian, which caused the null relationship in meta-analysis while there were significant effects in individual studies. Future studies should further explore the high heterogeneity that might be hidden in unmeasured gene–environment interactions, to explain the diverse findings among different populations.

## Abstract 2

Supplemental Digital Content is available in the text

## 1 Introduction

Chronic kidney disease (CKD) is an important issue in public health. The prevalence of CKD is approximately 10% around the world.^[1–3]^ CKD patients are at higher risk of cardiovascular diseases (CVDs) and all-cause mortality.^[4]^ Genetic factors such as ethnicity^[5]^ and familial inheritance^[6]^ play important roles in CKD. The heritability of CKD is 20% to 80%, and individual differences originate from genetic mutations.^[7–10]^ Genome-wide association studies have found that many polymorphisms cause CKD.^[11,12]^ Therefore, exploring CKD-related gene polymorphisms is important for reducing the burden of the disease.

*Methyltetrahydrofolate reductase* (*MTHFR*) is a key enzyme of 1-carbon cycle. It can convert inactive 5,10-methylenete tetrahydrofolate (THF) to active 5-methyl THF, which assists in converting cysteine to methionine. Homocysteine (Hcy), produced during the conversion process, is pro-inflammatory and causes vascular irritation, which increases the risk of atherosclerotic damage. The most common polymorphism in *MTHFR* is C677T (rs1801133). The C677T locates in exon 4 of *MTHFR* and causes a change from alanine to valine. A previous study found that this polymorphism changed the thermolabile of *MTHFR*^[13]^ and the concentration of homocysteine increased significantly.^[14]^

Although previous studies have reported an association between *MTHFR* C677T and CVD, a meta-analysis of randomized control trials (RCTs) found that folate supplements did not significantly decrease the risk of CVD.^[15]^ Many previous studies have researched the relationship between C677T and CKD but the results are inconsistent due to differences in ethnicity, lifestyle, and comorbidities among the study groups.^[16–22]^ Several meta-analyses have also explored the relationship between *MTHFR* C677T and diabetic nephropathy (DN) since 2007.^[23–31]^ However, no satisfactory consensus has been reached. Therefore, we applied a trial sequential analysis (TSA) proposed by Thorlund et al^[32]^ to evaluate whether the most recent conclusions were supported by the current cumulative samples.

Although the meta-analysis had a higher sample size compared with the traditional case–control study, the population heterogeneity may cause meaningless result. This was mostly due to potential gene–environment interactions. Gene–environment interactions had not yet been explored in conventional meta-analyses owing to the difficulty in using summary data to analyze interactions. For this study, we developed a revised version of meta-regression, case-weighted meta-regression, which resolved the problem.^[33]^ This could be applied in the relationship between *MTHFR* C677T and CKD to explore the heterogeneity.

The aim of this study was to evaluate whether the latest conclusions are supported by the current cumulative samples using trial sequential analysis (TSA). We also provided additional case–control samples to enhance the current evidence. Moreover, we applied case-weighted meta-regression to explore potential gene–environment interactions.

## 2 Materials and methods

### 2.1 Case–control study

#### 2.1.1 Ethical considerations

This study was approved by the institutional review board (TSGH-1-104-05-006) of the Tri-Service General Hospital (TSGH). Volunteers signed the consent form after the investigators had provided an explanation of the study.

#### 2.1.2 Subjects

Subjects in the case group were hemodialysis patients from 7 Taipei Dialysis Centers during 2015 to 2017. The exclusion criteria were as following:

(1) dialysis period of < 3 months,
(2) presence of cancers, and
(3) insufficient blood samples.

A total of 882 cases were included in the analysis.

Subjects in the control group were selected from volunteers who participated in a physical exam at the Health Management Centre at TSGH. The serum creatinine levels of volunteers were tested, and the Modification of Diet in Renal Disease (MDRD) formula^[34]^ was used to calculate the estimated glomerular filtration rate (eGFR). The exclusion criteria were shown as following:

(1) eGFR < 60 ml/min/1.73 m^2^,
(2) presence of kidney-related diseases (such as positive proteinuria),
(3) presence of cancers, and
(4) insufficient blood samples.

Finally, 755 subjects were included in the control group for the analysis.

Demographic data including age, sex, diabetes, hypertension, body mass index (BMI) and blood biochemical parameters (blood urea nitrogen, creatinine, triglycerides, cholesterol, and eGFR) were collected via questionnaire and medical records.

#### 2.1.3 Genomic DNA extraction and genotyping

Medical technologists or nurses collected 5 mL of intravenous blood samples from each of the volunteers. Genomic DNA from peripheral blood samples was isolated using standard procedures for proteinase K (Invitrogen, Carlsbad, CA) digestion and the phenol/chloroform method.^[35]^*MTHFR* C677T was genotyped by iPLEX Gold SNP genotyping.^[36]^ Inter- and intra-replication validation was used to assess the genotyping experiment quality. Inter-replication validation was repeated for 78 samples (∼5%), and the concordance rate was 100%.

#### 2.1.4 Statistical analysis

Continuous variables of the general demographic data were expressed as mean and standard deviation using Student *t* test. The control group was tested for representativeness using the Hardy–Weinberg equilibrium (HWE) test.^[37]^ Differences in genotype and allele frequencies between hemodialysis patients and healthy controls were tested using a χ^2^ test or Fisher exact test. ORs and 95% confidence intervals (CIs) for the risk of end-stage renal disease (ESRD) were calculated using logistic regression. Calculation of genetic polymorphism and ESRD risk was expressed using allele type, genotype, and dominant/recessive models. A p-value of < 0.05 was considered significant, and the Bonferroni correction was used for multiple comparison correction. R 3.4.2 (R Project for Statistical Computing, Vienna, Austria) was used for statistical analyses.

### 2.2 Meta-analysis

#### 2.2.1 Search methods and criteria for study consideration

The PRISMA checklist and Meta-analysis on Genetic Association Studies Checklist are described in Supplemental Digital Content (S1 Table).^[38]^ Related terms of “*MTHFR* C677T” and “chronic kidney disease” were used to search the PubMed, EMBASE, and Web of Science databases for articles published up to December 31, 2018 Supplemental Digital Content (S2 Table). The language of the articles was limited to English. In addition, the publications included in the meta-analysis studies were manually examined to avoid the omission of important articles. The inclusion criteria were as following:

(1) the study design was case–control or cross-sectional;
(2) CKD was defined according to the National Kidney Foundation as kidney damage by clinical diagnosis or a GFR < 60 mL/min/1.73 m^2^ (samples with lupus nephritis, polycystic kidney disease, endemic nephropathy, and reflux nephropathy were excluded);
(3) subjects in the control group had normal renal function;
(4) the article contained the detailed genetic distribution of the *MTHFR* C667T; and
(5) the samples were aged >18 years.

When the proportion of diabetic subjects in the case group in an article was 100%, the control group was selected from diabetic patients.

#### 2.2.2 Data extraction

Two reviewers (G-rC and CL) independently extracted the literature data. The collected data included the last name of the first author, year of publication, country, race of the study population, male proportion, mean BMI, diabetes prevalence, hypertension prevalence, smoking prevalence, and gene distribution in the case and control groups.

#### 2.2.3 Statistical analysis

The data of all included articles were described using proportions or mean values where appropriate. The meta-analysis used ORs with 95% CI to examine the correlation between *MTHFR* C677T polymorphisms and CKD. I^2^ calculated with the Cochrane Q test was used to assess heterogeneity. I^2^ > 50% indicated moderate-to-high heterogeneity.^[39]^ Egger regression and funnel plot were used to examine the symmetry after combination. Genetic models including allele type, and dominant and recessive models were used to calculate *MTHFR* C677T polymorphisms and risk of CKD. The random effects model was used to combine results.

Two methods were used to explore the moderator effects in previous meta-analyses:

(1) dividing different studies into high- and low-risk groups according to the distribution of moderators for subgroup analysis; and
(2) applying meta-regression to analyze the correlation between effect size and the distribution of moderators.

In the first method, it is difficult to determine the cutoff point for the moderator, and this tends to increase the probability of false positives.^[40,41]^ Therefore, meta-regression is usually used for moderator effect analysis. However, the matched studies (such as gender- and age-matched studies) were the necessary condition to use traditional meta-regression for analyzing moderator effects. Lin et al^[33]^ have developed a revised version of meta-regression, known as case-weighted meta-regression, to break this limitation. Under the 2 assumptions, rare disease and independence between moderator and independent variable, the odds ratio (OR) of exposure to the disease tended to be close to the proportion of moderators in the case group. Therefore, it was better to extract data from the case group during the meta-analysis.

Considering the high heterogeneity of the combined results, case-weighted meta-regression was applied to examine the source of heterogeneity in this study. Possible environmental factors (such as ethnicity, sex, BMI, and diabetes) were included, and summary data from the case group were extracted to explore gene–environment interactions. The significance level of this study was set at 0.05. The “metafor"^[42]^ and “meta”^[43]^ packages of R software version 3.4.2 were used. TSA was used to validate whether the meta-analysis results obtained a decisive conclusion.^[32]^ OR was used as an effect measure, and the random effects model was used to combine results. Zero event handling was set at 1. The degree of freedom was set at 2. TSA was used for stratification analysis based on race (Caucasian and Asian). Type 1 error was set at 0.05. Power was set at 0.8. Heterogeneity was set at 80%. A review of past literature showed that the OR of correlation between *MTHFR* C677T and CKD was around 1.2. The 1000 Genome database was used as a reference for minor allele frequency, which is 0.30 for Asians and 0.36 for Caucasians.^[44]^

## 3 Results

### 3.1 Case–control study

Table 1 shows the distribution of general demographic variables and blood biochemical parameters of the study population. A total of 1637 subjects were enrolled in this study, including 755 subjects in the control group with a mean age of 73.86 ± 7.16 years (312 males and 443 females) and 882 subjects in the case group with a mean age of 71.23 ± 13.22 years (461 males and 421 females). There were 607 non-diabetic ESRD patients in the case group with a mean age of 75.13 ± 12.02. The proportion of males was lower in the control group than in the case group (*P* < .001), and the mean age in the control group was higher than that in the case group (*P* = .006). The case group showed a higher proportion of diabetic patients, a higher proportion of hypertensive patients, higher creatinine levels, higher fasting blood glucose levels, and higher triglyceride levels than the control group. In addition, the case group showed lower cholesterol level. Table 2 shows the distribution differences in *MTHFR* C677T genotype between the case and control groups. The gene frequency of the control group followed the Hardy–Weinberg equilibrium (*P* = .493). The distribution of the T allele in the control and case groups was 30.5% and 25.9%, respectively, and this difference was significant (*P* = .004).

**Table 1:**
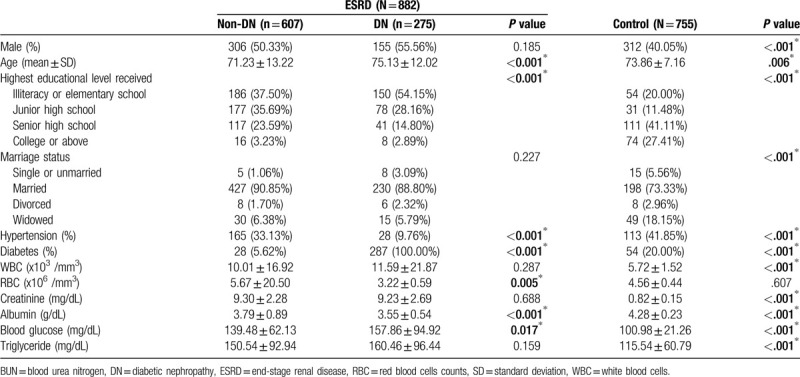
Demographic characteristic and laboratory data of case group and control group.

**Table 2:**
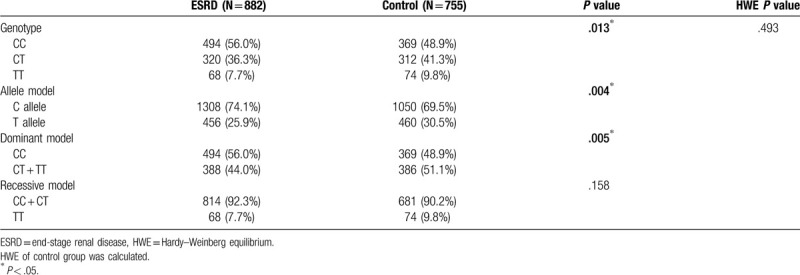
MTHFR C677T genotype distribution in case group and control group.

In the allele model after adjusting for sex, age, history of hypertension, and history of diabetes, the T allele of *MTHFR* C677T was a significant protective factor for ESRD [OR: 0.760 (95% CI: 0.639–0.904)] (Table 3). Similar results were obtained in the genotype and dominant models. To enhance the level of evidence, the case–control data were included in the meta-analysis, and TSA was applied to validate whether the most recent conclusion is supported by the current cumulative samples.

**Table 3:**
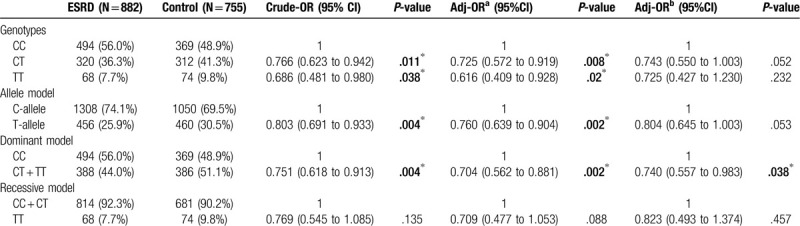
Logistic regression for MTHFR C677T and ESRD using different analytic model.

### 3.2 Meta-analysis

**Figure 1:**
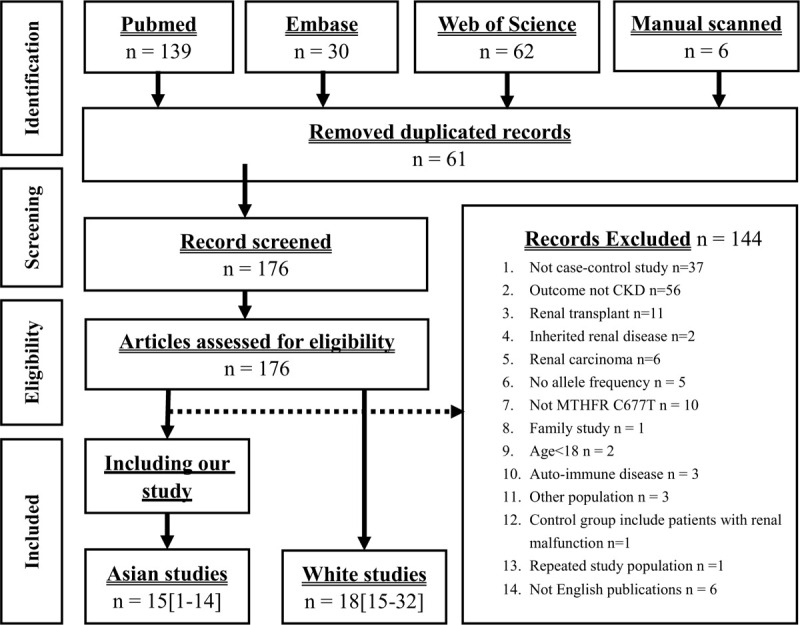
PRISMA flow chart of meta-analysis. Flow chart of the identification process for eligible studies.

**Figure 2:**
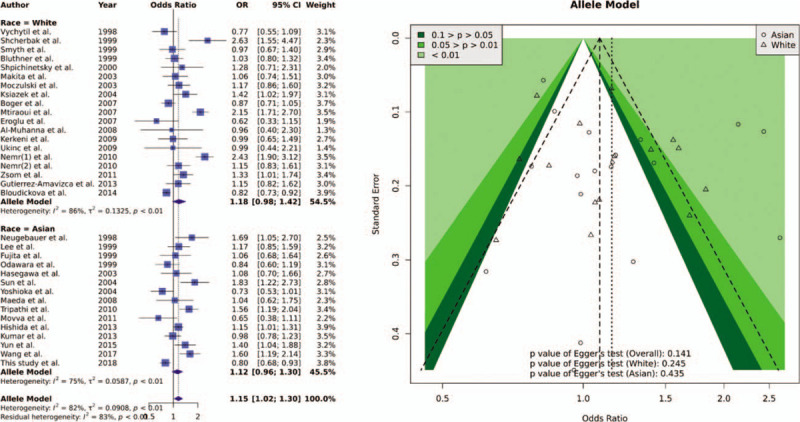
Forest plot and funnel plot of association between MTHFR C677T and all-cause CKD in allele model. Forest plot of MTHFR C677T and all-cause CKD stratified into different races (Allele model).

The study identification process is shown in Fig. 1. In meta-analysis, a total of 231 articles were found from PubMed, EMBASE, and Web of Science. Moreover, 6 articles were manually added from previous meta-analyses. Excluded were: 61 repeated articles, 37 non-case–control studies, 56 articles without CKD, 11 articles focused on kidney transplantation patients, 8 articles for hereditary kidney disease,^[45–60]^ 5 articles that did not provide complete genotype information,^[22,61–64]^ 10 articles without *MTHFR* C677T,^[65–74]^ 1 familial study,^[75]^ 2 articles on minors,^[76,77]^ 3 articles for auto-immune diseases,^[78–80]^ 3 articles covering non-Asian and non-Caucasians,^[81–83]^ 1 article with an incorrect definition of a control group,^[84]^ 1 article using a duplicated population,^[18]^ and 6 non-English articles.^[85–90]^ Finally, 33 papers were included for meta-analysis. The study by Nemr et al^[91]^ included 2 populations, which resulted in 34 populations being added to the present study Supplemental Digital Content (S3 Table). The allele model was used to combine 34 populations, and the results showed significant differences [OR: 1.15 (95% CI: 1.02–1.30)] and high heterogeneity (I^2^ = 82%). However, the stratified analyses showed null associations in Asian [OR: 1.12 (95% CI: 0.96–1.30)] and Caucasian [OR: 1.18 (95% CI: 0.98–1.42)], respectively. No significant asymmetry was discovered between the articles. The forest and funnel plots are shown in Fig. 2.

### 3.3 TSA evaluation

**Figure 3:**
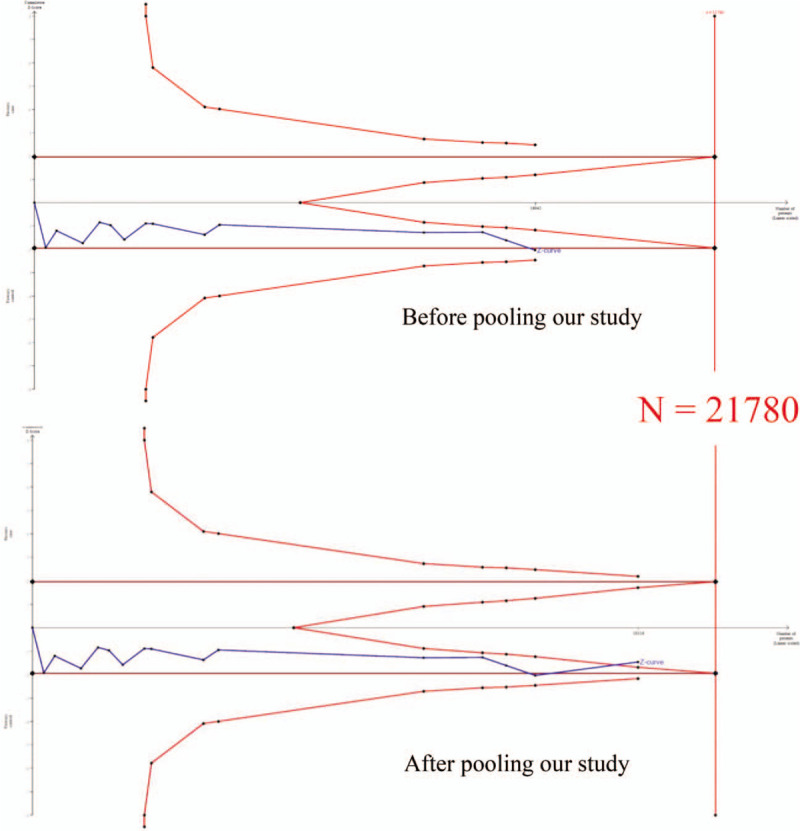
Trial sequential analysis of MTHFR C677T and all cause CKD among Asian population. TSA is a methodology that calculates sample size for meta-analysis with correction of statistical significance. We performed a TSA under allele model assumption but replaced the allele count with the sample size (divided by 2). Bold line represents cumulative sample size of our case control study. Detailed settings: Significance level = 0.05; Power = 0.80; hypothetical proportion of T allele in control = 0.30; least extreme OR to be detected = 1.2; I2 (heterogeneity) = 80%.

In the TSA evaluation, the cumulative sample size for Caucasians exceeded the threshold value Supplemental Digital Content (S1 Fig), showing that *MTHFR* C677T and all-cause CKD are not significantly related (allele model), and a decisive conclusion could be validated. Before adding our case–control samples, the cumulative sample size for the Asian population was 8021 (Fig. 3). After adding our case–control samples, the cumulative sample size was 9658, and the Z curve reached the futility area. This result shows that *MTHFR* C677T and all-cause CKD are also not significantly related in Asian, and a decisive conclusion could be confirmed. Thus, the case–control samples in this study provided critical evidence for establishing a decisive conclusion.

### 3.4 Gene–environment interactions

The high heterogeneity (I^2^ = 75%) in Asian could explain the contradictory results between the case–control study and the meta-analysis. The overall analysis had shown a significant association, but stratified analyses showed a non-significant association in both the Asian and the Caucasian sample groups. Therefore, environmental moderators should be explored to explain the heterogeneity. Table 4 summarizes the moderator effects in the association between *MTHFR* C677T and CKD (allele model). Case-weighted meta-regression was used to analyze the race, sex, BMI, hypertension, DM, and smoke. However, there was no significant gene-environmental interaction between all measured factors and *MTHFR* C677T.

**Table 4:**
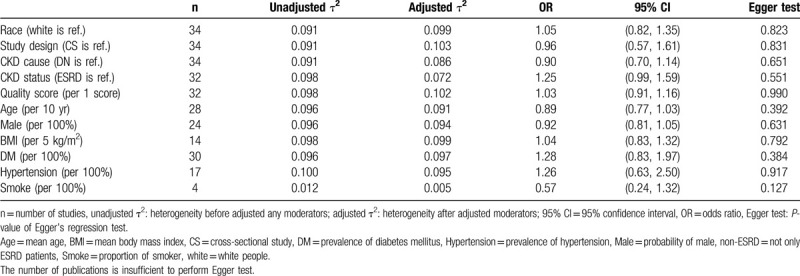
Moderator effects of allele type model [T vs C] (all-cause CKD).

## 4 Discussion

In summary, this meta-analysis showed that *MTHFR* C677T is not significantly correlated with all-cause CKD in Asian. TSA results showed that the current cumulative samples were sufficient to reach a decisive conclusion and our case–control sample was the critical evidence. The meta-analysis also showed that *MTHFR* C677T is not significantly correlated with all-cause CKD in Caucasian, and the TSA results showed that the cumulative sample size was also sufficient to reach a decisive conclusion. To the best of our knowledge, this study is the first meta-analysis to explore the correlation between *MTHFR* C677T and all-cause CKD using TSA.

Hcy is mainly metabolized by the liver and kidneys. Due to poor renal function in CKD patients, they are unable to metabolize Hcy, which consequently accumulates in high concentrations.^[92]^ Hcy and its derivatives activate platelets and increase coagulation and oxidative stress. Therefore, they cause vascular endothelial damage and smooth muscle cell hyperplasia,^[93–96]^ thereby increasing the risk of vascular and organ damage. The risk of stroke, atherosclerosis, ischemic heart disease, and death is higher in CKD patients than in the general population,^[97–99]^ and previous studies have speculated that Hcy plays an important role in these diseases. Many observational studies have found that the higher the Hcy level, the greater the risk of CVD-related disease and mortality in CKD patients.^[100]^ However, RCTs have found that the use of folate supplements to reduce Hcy concentration did not lower CVD risk.^[101–103]^ Moreover, *MTHFR* C677T has been shown to reduce *MTHFR* activity and increase *in vivo* Hcy concentrations.^[13]^ Additional pathological and physiological studies are required to investigate the relationship between renal function and *MTHFR* C677T.

The case–control study found T allele is a significant protective factor for ESRD after adjusting for age, sex, comorbidities, and other confounding factors. Although these results are consistent with those reported by of Bloudickova et al,^[104]^ other studies have shown that T allele is not correlated^[105–109]^ and is not a significant risk factor.^[110]^ In addition to differences in ethnicity and region, different demographic characteristics may also result in these contradictions. In our meta-analysis, after adding our case–control samples to the Asian population, the TSA result showed that *MTHFR* C677T was not significantly correlated with CKD and the cumulative sample size reached the futility area, indicating that a definite conclusion could be reached and that the case–control sample was crucial. The high heterogeneity (I^2^ = 75%) in the Asian sample could explain the contradictory results between the case–control study and the meta-analysis.

The inconsistent result between proposed homocysteine related mechanism and our non-significant result might be explained by missing heritability issue. The term “missing heritability” was first proposed by Manolio in 2009.^[111]^ Even we had conducted the genome wide association study on a lot of complex phenotype such as height, diabetes, and cholesterol, we still only can find a small part of significant polymorphism and just can explain a little part of each phenotype. This phenomenon is still not solved now in complex phenotype. Chronic kidney disease is clearly a complex disease, which might be affected by multiple factors including ethnicity^[112]^ and family history of disease.^[113]^ In the best of our knowledge, the genome wide association study with large sample also only explained 2% variance in chronic kidney disease.^[12]^ Therefore, the non-significant finding was common. The significant findings in individual studies but non-significant results in larger study might be due to the lack of control for population stratification bias.^[114]^ The high study heterogeneity of our meta-analysis might be due to this issue, and we considered the studies using mixture population was not suitable. We considered the future study should be conducted by whole genome survey process and use the related method for correcting this issue.

This meta-analysis did not detect significant interactions between the measured environmental factors and *MTHFR* C677T, suggesting that there may be further unmeasured factors to be discovered. Additional studies exploring the effects of gene–environment interactions on CKD are warranted. Ma et al^[115]^ proposed a significant interaction between *MTHFR* C677T and smoking in people with type 2 diabetes or DN. Among those who smoked, the risk of DN was higher in subjects with the T allele (OR = 1.6, *P* = .006). Another large-scale cross-sectional study in Japan found no significant interaction between *MTHFR* C677T and blood folate concentration on CKD.^[116]^ Further stratified analysis found that the risk of developing CKD was higher in subjects with the TT genotype and low blood folate concentration (OR = 2.07, 95% CI = 1.30–3.31). Regarding drug–gene interactions, many RCTs have showed significant interactions between *MTHFR* C677T and folate supplements.^[117]^ In subjects with the TT genotype, the Hcy reduction effects of folate supplements were significant, although a meta-analysis of large-scale RCTs denied the presence of such interactions.^[118]^

In addition to gene–environment interactions, other studies have explored the effects on the risk of CKD of gene–gene interactions. Rahimi et al^[119]^ found that polymorphism at *MTHFR* C677T and another locus (A1298C) increased risks of micro-albuminuria and macro-albuminuria (OR_Micro_ = 4.32, 95% CI = 1.5–12.6; OR_Macro_ = 20.4, 95% CI = 5.3–79). Jafari et al^[64]^ found significant interactions between endothelial nitric oxide synthase (eNOS) G894T and *MTHFR* C677T in type 2 diabetes, and where both T allele existed, the risk of developing macro-albuminuria was higher (OR_Macro_ = 38.5, 95% CI = 4.7–319). eNOS encodes nitric oxide synthase, which plays an important role in regulating vascular endothelial function. *MTHFR* C677T polymorphism increases Hcy concentration and disrupts vascular endothelial cell function. Therefore, we suggest further studies investigate the gene–gene interaction between these 2 genetic factors and *MTHFR* C677T. However, the 2 aforementioned articles had low sample sizes, and a larger one may be required to validate these interactions.

Our study has the following strengths. To begin with, we included 33 English articles regarding the correlation between *MTHFR* C677T and CKD in PubMed, EMBASE, and Web of Science (including this study) databases and included 19,734 subjects. In addition, TSA analysis found that a decisive conclusion could be established and the samples in this study were critical evidence. Secondly, due to limitations in summary data for conventional meta-regression analysis, sex, age, and other variables could not be used for interaction analysis. In this study, case-weighted meta-regression was firstly applied for interaction analysis, which not only solved the problem of insufficient power in individual articles but also detected potential interactions in meta-analysis. Thirdly, the Newcastle–Ottawa Scale was used to perform a complete article quality evaluation, and whether article quality affected the combined results was investigated. Although there was no effect of this factor on the combined results, this approach enhances the reliability of this study. Finally, our meta-analysis could prevent errors compared with previous studies. Our study excluded articles with incomplete genotype information and used the allele model to combine results. Egger regression was not significant for Asians and Caucasians (*P* > .05). In previous meta-analyses, the fixed effects model was used for combination if heterogeneity was not significant (*P* ≥ .1) and the random effects model was used if heterogeneity was significant (*P* < .1).^[120–125]^ This study only used the random effect model to combine results, and could avoid the serious bias that can be caused when heterogeneity is used to select a model.^[126]^

This study has 3 limitations. First, because individual data could not be obtained in the meta-analysis, the possibility of ecological fallacy cannot be excluded. Although this is a limitation of the meta-analysis, many variables were included in this study and the correlation between these variables was explored. Therefore, the possibility that the study conclusion will be affected by ecological fallacy is extremely low. Second, data on the diet and folate intake status are limited. Therefore, the potential effect of folic acid intake on the association between *MTHFR* C677T and CKD could not be explored. Third, many of the articles did not provide the complete distribution status of the moderators (such as the mean age and male proportion of populations), which decreased the powers. We suggest future case–control studies should provide detailed information on these factors.

## 5 Conclusions

In conclusion, high heterogeneities were found in both Caucasian and Asian, which produced a null relationship in meta-analysis but significant effects in individual studies. However, after using the case-weighted meta-regression, all the measured factors still cannot explain the source of heterogeneity. Future studies should further explore the high heterogeneity, which might be hidden in unmeasured gene–environment interactions, to explain the diversity findings among different populations. The gene–gene interactions should also be considered such as eNOS G894T and *MTHFR* A1298C. More specific stratified analysis based on heterogeneity may help us to understand the real relationship between *MTHFR* C677T and CKD.

## Author contributions

H-L C, M-C T, C-C K, W-T C, S-L S conceived and designed the experiments. H-L C, W-T C, H S performed the experiments. H-L C and D-JT analyzed the data. H-L C, P-J H, C-C C, S-L S provided the reagents, materials, and analysis tools. H-L C and G-R C wrote the paper. H-L C, D-J T, Y-H C, W-T C, S-L S were responsible for the critical review and providing comments. H-L C, S-LS and D-JT were responsible for modifying the manuscript.

## Supplementary Material

Supplemental Digital Content

## Supplementary Material

Supplemental Digital Content

## Supplementary Material

Supplemental Digital Content

## Supplementary Material

Supplemental Digital Content
