# Supplementary figures and images for: Decisive evidence corroborates a null relationship between MTHFR C677T and chronic kidney disease: A case–control study and a meta-analysis
Source: Medicine (Baltimore). 2020 Jul 17;99(29):e21045. doi: 10.1097/MD.0000000000021045 (PMC7373545; doi:10.1097/MD.0000000000021045)

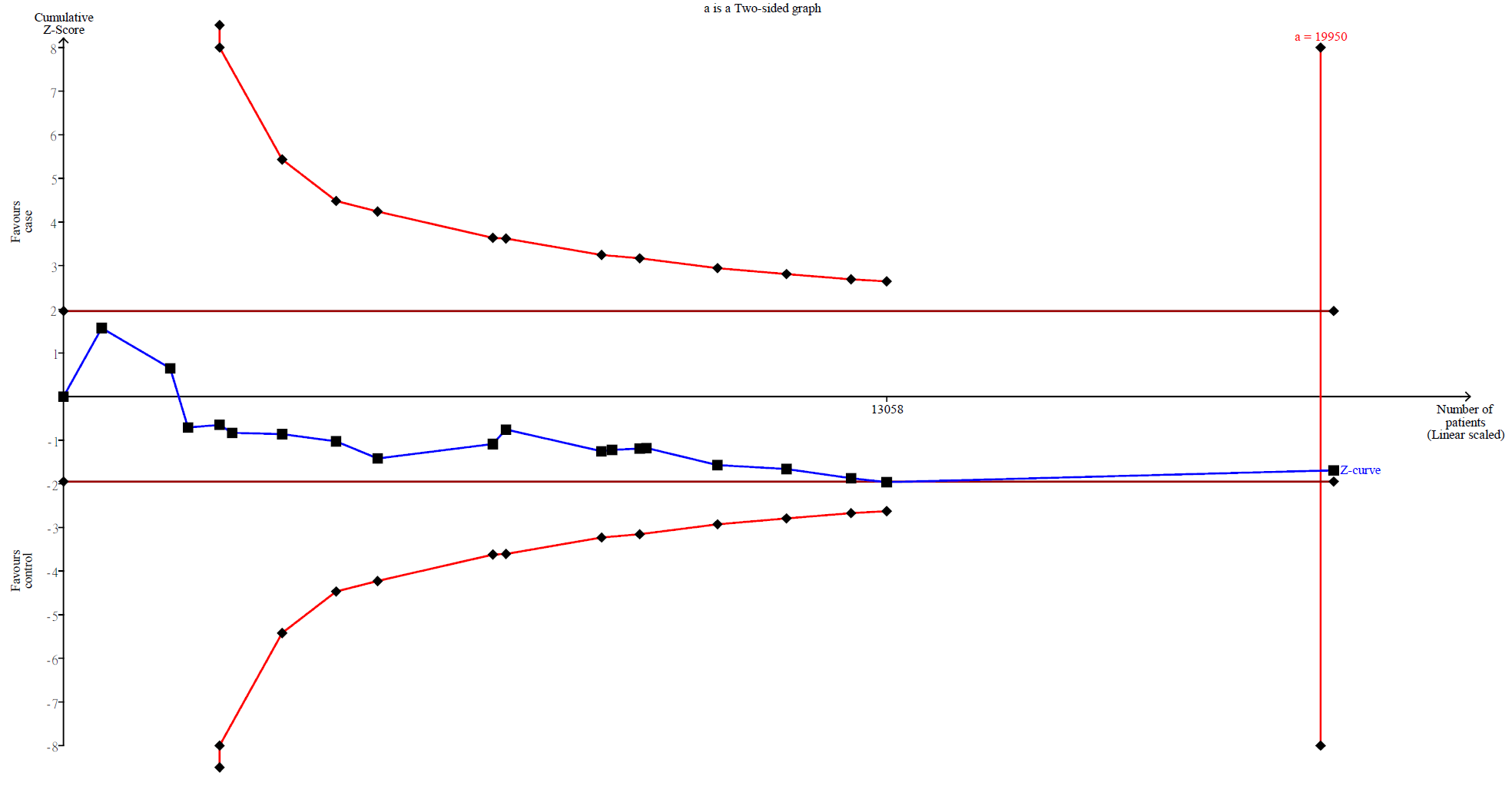


N = 9975

**Figure S3 Trial Sequential Analysis of MTHFR C677T and all cause CKD among White population**

Supplement: Supplemental Digital Content [file medi-99-e21045-s001.docx]
