# Supplementary material for: Decisive evidence corroborates a null relationship between MTHFR C677T and chronic kidney disease: A case–control study and a meta-analysis
Source: Medicine (Baltimore). 2020 Jul 17;99(29):e21045. doi: 10.1097/MD.0000000000021045 (PMC7373545; doi:10.1097/MD.0000000000021045)
Supplement: Supplemental Digital Content [file medi-99-e21045-s003.docx]

**Table S2 MESH Term Search strategies for Meta-analysis**

| **Relevant text of MTHFR C677T**   1. MTHFR 2. Methylenetetrahydrofolate Reductase (NADPH) 3. Methylene-THF Reductase (NADPH) 4. Methylenetetrahydrofolate Reductase 5. 5,10-Methylenetetrahydrofolate Reductase (NADPH) 6. Methylene Tetrahydrofolate Reductase 7. Tetrahydrofolate Reductase, Methylene 8. Polymorphisms, Genetic 9. Genetic Polymorphisms 10. Polymorphism (Genetics) 11. SNPs 12. Single Nucleotide Polymorphism 13. Mutation, Point 14. C677T 15. rs1801133 16. Ala222Val 17. (1 or 2 or 3 or 4 or 5 or 6 or 7) and (8 or 9 or 10 or 11 or 12 or 13) and(14 or 15 or 16 )   **Relevant text of chronic kidney disease**   1. Renal Insufficiency, Chronic 2. Chronic kidney disease 3. Chronic Kidney Insufficiency 4. Chronic Renal Diseases 5. Chronic Renal Insufficiency | 1. Kidney Insufficiency, Chronic 2. CKD 3. Kidney Failure, Chronic 4. Chronic Kidney Failure 5. End-Stage Kidney Disease 6. End-Stage Renal Disease 7. End-Stage Renal Failure 8. Renal Disease, End-Stage 9. Renal Failure, Chronic 10. Renal Failure, End-Stage 11. ESKD 12. ESRD 13. ESRF 14. Proteinuria 15. Albuminuria 16. Nephropathy 17. Nephritis 18. Dialysis 19. Glomerular filtration rate 20. GFR 21. 24 or 25 or 26 or 27 or 28 or 29 or 30 or 31 or 32 or 33 or 34 or 35 or 36 or 37 or 38 or 39 or 40 or 41 or 42   **Combined (Final strategy)**   1. 17 and 43 |
| --- | --- |

**MeSH Browser**: <http://www.nlm.nih.gov/mesh/MBrowser.html>

**PubMed**: <http://www.ncbi.nlm.nih.gov/pubmed>

**Cochrane Library**: <http://www.thecochranelibrary.com>

**Embase**: https://www.embase.com

**Web of Science**: https://apps.webofknowledge.com
