# Supplementary material for: Decisive evidence corroborates a null relationship between MTHFR C677T and chronic kidney disease: A case–control study and a meta-analysis
Source: Medicine (Baltimore). 2020 Jul 17;99(29):e21045. doi: 10.1097/MD.0000000000021045 (PMC7373545; doi:10.1097/MD.0000000000021045)
Supplement: Supplemental Digital Content [file medi-99-e21045-s004.docx]

| **Table S3 Characteristics of published studies included in this meta-analysis.** | | | | | |
| --- | --- | --- | --- | --- | --- |
| **Author, year** | **Race** | **Study design** | **CKD cause** | **Kidney function of case** | **Definition of case** |
| Vychytil et al., 1998[[272](#_ENREF_272)] | White | CC | Mixed | ESRD | Peritoneal dialysis |
| Neugebauer et al., 1998[[273](#_ENREF_273)] | Asian | CC | DN | Non-ESRD | microalbuminuria (23 to 230 mg albumin/mmol creatinine) |
| Lee et al., 1999[[275](#_ENREF_275)] | Asian | CC | Mixed | ESRD | Hemodialysis |
| Bluthner et al., 1999[[260](#_ENREF_260)] | White | CC | DN | non-ESRD | UAE>20ug/min |
| Fujita et al., 1999[[250](#_ENREF_250)] | Asian | CC | DN | non-ESRD | UAE>200ug/min |
| Odawara et al., 1999[[276](#_ENREF_276)] | Asian | CC | DN | non-ESRD | Not reported |
| Shcherbak et al., 1999[[274](#_ENREF_274)] | White | CC | DN | non-ESRD | albumin excretion >300 mg/daily |
| Smyth et al., 1999[[268](#_ENREF_268)] | White | CC | DN | non-ESRD | persistent proteinuria (>500 mg/24 h) |
| Shpichinetsky et al., 2000[[277](#_ENREF_277)] | White | CC | DN | non-ESRD | Persistent micro- or macroalbuminuria (>30 mg/24 h) |
| Hasegawa et al., 2002[[251](#_ENREF_251)] | Asian | CC | DN | ESRD | Hemodialysis |
| Makita et al., 2003[[263](#_ENREF_263)] | White | CC | DN | ESRD | medical history, medical records |
| Moczulski et al., 2003 | White | CC | DN | non-ESRD | ACR >28.2 for men and >40.2 for women |
| Sun et al., 2004[[278](#_ENREF_278)] | Asian | CC | DN | non-ESRD | UAE> 200ug/min |
| Ksiazek et al., 2004[[262](#_ENREF_262)] | White | CC | DN | non-ESRD | UAE > 300 mg/24 h |
| Yoshioka et al., 2004[[257](#_ENREF_257)] | Asian | CC | DN | non-ESRD | ACR >30 mg/g |
| Boger et al., 2007[[30](#_ENREF_30)] | White | CC | DN | ESRD | Standardized questionnaire and reviewing patients’ medical record |
| Mtiraoui et al., 2007[[279](#_ENREF_279)] | White | CC | DN | non-ESRD | UAE of >30 mg/24hr and/or Scr >176 mmol/L |
| Eroglu et al., 2007[[280](#_ENREF_280)] | White | CC | DN | non-ESRD | Not reported |
| Al-Muhanna et al., 2008[[259](#_ENREF_259)] | White | CC | Mixed | ESRD | Hemodialysis |
| Maeda et al., 2008[[281](#_ENREF_281)] | Asian | CS | DN | non-ESRD | ACR>30 mg/g |
| Kerkeni et al., 2009[[261](#_ENREF_261)] | White | CC | Mixed | non-ESRD | Not reported |
| Ukinc et al., 2009[[282](#_ENREF_282)] | White | CC | DN | non-ESRD | overt nephropathy |
| Tripathi et al., 2010[[283](#_ENREF_283)] | Asian | CC | Mixed | ESRD | Hemodialysis |
| Nemr et al., 2010[[284](#_ENREF_284)] | White | CC | DN | non-ESRD | UAE of > 30 mg/24 h, and/or increased creatinine > 176 mmol/ L |
| Movva et al., 2011[[285](#_ENREF_285)] | Asian | CC | DN | non-ESRD | Microalbuminuria UAE: 30 to 299 mg/day, Overt nephropathy UAE>300 mg/day. |
| Zsom et al., 2011[[26](#_ENREF_26)] | White | CC | Mixed | non-ESRD | renal biopsy, medical history, renal ultrasound or CT scan |
| Kumar et al., 2013[[286](#_ENREF_286)] | Asian | CC | DN | non-ESRD | proteinuria >500 mg/day |
| Gutierrez-Amavizca et al., 2013[[109](#_ENREF_109)] | White | CC | Mixed | ESRD | Hemodialysis |
| Hishida et al., 2013[[110](#_ENREF_110)] | Asian | CS | Mixed | non-ESRD | eGFR <60 ml/min/1.73 m2 |
| Bloudickova et al., 2014[[114](#_ENREF_114)] | White | CC | Mixed | ESRD | Hemodialysis |
| Yun et al., 2015[[287](#_ENREF_287)] | Asian | CC | HN | non-ESRD | ACR between 30 and 300 mg/g |
| Wang et al., 2017[[113](#_ENREF_113)] | Asian | CC | DN | non-ESRD | Peritoneal dialysis |
| This study | Asian | CC | Mixed | ESRD | Hemodialysis |
| CC: case control study; CS: cross-sectional survey; DN: diabetic nephropathy; HN: hypertensive nephropathy; ESRD: only ESRD patients; non-ESRD: not only ESRD patients; UAE: urinary albumin excretion rate; ACR: Albumin creatinine ratio; eGFR: estimated glomerular filtration rate; RRT: renal replacement therapy; CT: computed tomography; SCr: serum creatinine. | | | | | |
